# Yoga and Mindfulness-Based Rehabilitation After Myocardial Infarction: A Systematic Review

**DOI:** 10.3390/healthcare14081106

**Published:** 2026-04-21

**Authors:** Chiara Bianchi, Laura Rotondo, Claudio Bersani, Rita Pavasini, Federico Marchini, Serena Caglioni, Andrea Raisi, Gianluca Campo, Elisabetta Tonet

**Affiliations:** 1 Cardiology Unit, Azienda Ospedaliero Universitaria of Ferrara, Via Aldo Moro 8, 44124 Ferrara, Italy; chiara02.bianchi@edu.unife.it (C.B.); laura.rotondo11@gmail.com (L.R.); claudio.bersani@edu.unife.it (C.B.); pvsrti@unife.it (R.P.); mrcfrc2@unife.it (F.M.); serenacaglioni@gmail.com (S.C.); cmpglc@unife.it (G.C.); 2 Center for Exercise Science and Sport, University of Ferrara, Via Gramicia 35, 44121 Ferrara, Italy; andrea.raisi@unife.it

**Keywords:** yoga, mindfulness, myocardial infarction, lifestyle, rehabilitation

## Abstract

**Highlights:**

**What are the main findings?**

- The available studies about yoga and mindfulness in patients with myocardial infarction show consistent improvements in psychological well-being, including reduced stress, anxiety, and depressive symptoms, together with better quality of life.
- Some evidence also suggests benefits for physical recovery, such as improved exercise tolerance and favorable changes in body weight and heart-related risk factors.
- These interventions were found to be safe, well accepted by patients, and easy to implement in different healthcare settings.

**What are the implications of the main finding?**

- The findings suggest that yoga and mindfulness may meaningfully complement standard rehabilitation. When used alongside conventional medical care, they may help promote a more holistic and patient-centered recovery after a heart attack.

**Abstract:**

**Background:** Psychological distress, autonomic dysregulation, and unhealthy lifestyle behaviors are common after myocardial infarction (MI) and negatively affect cardiovascular outcomes. In recent years, integrative mind–body interventions, such as yoga and mindfulness-based approaches, have gained increasing attention as adjuncts to conventional cardiac rehabilitation (CR) programs. However, evidence regarding their effectiveness in post-MI populations remains fragmented. **Objective:** This systematic review aimed to synthesize the available evidence on the effects of yoga- and mindfulness-based interventions in patients following myocardial infarction. **Methods:** A systematic literature search was conducted across major electronic databases to identify randomized controlled trials and observational studies evaluating yoga- or mindfulness-based interventions in post-MI patients. Eligible studies included adult MI populations. Study selection and quality assessment were performed according to predefined criteria. **Results:** The 10 included studies suggest that yoga-based cardiac rehabilitation programs may provide benefits beyond standard care, particularly in terms of self-rated health, psychological well-being, and return to pre-infarction daily activities. Mindfulness-based interventions were associated with reductions in anxiety and perceived stress, improvements in blood pressure control, enhanced social support, and better health-related quality of life. Several studies also reported favorable effects on autonomic balance and stress-related physiological markers. Finally, a study reported benefits in terms of MACE (*p* = 0.032). However, heterogeneity in intervention protocols, outcome measures, and study designs limited direct comparisons across studies. **Conclusions:** Yoga and mindfulness-based interventions appear to be promising complementary strategies in post-MI care.

## 1. Introduction

Myocardial infarction (MI), commonly known as a heart attack, is defined as myocardial necrosis caused by acute ischemia, typically resulting from obstruction of a coronary artery [1,2]. MI remains one of the leading causes of cardiovascular morbidity and mortality worldwide, and comprehensive cardiac rehabilitation (CR) represents a cornerstone of secondary prevention. Contemporary CR programs adopt a multidomain approach, integrating supervised exercise training, cardiovascular risk factor modification, optimization of medical therapy, and dietary counseling [3]. Despite these well-established components, a substantial proportion of MI survivors continue to experience psychological distress, autonomic imbalance, and suboptimal lifestyle behaviors, all of which may adversely influence cardiovascular outcomes. In recent years, increasing attention has been directed toward integrative mind–body approaches, such as yoga and mindfulness, as potential adjuncts to conventional CR. These interventions specifically target psychological and autonomic pathways that are not fully addressed by traditional rehabilitation strategies. Yoga is a mind–body discipline that integrates physical postures (asanas), breathing techniques (pranayama), and meditation. The World Health Organization (WHO) recognizes yoga as an intervention capable of reducing stress and anxiety, promoting relaxation and self-awareness, and improving physical well-being through enhanced muscle strength and flexibility [4]. Accordingly, yoga has gained growing interest as an integrative therapeutic strategy in the prevention and management of chronic diseases, including cardiovascular disorders. Evidence from the literature suggests that yoga-based CR programs may provide benefits beyond standard care in patients with MI, particularly with respect to self-rated health and return to pre-infarction daily activities [5,6]. Short-term interventions have also demonstrated favorable physiological effects, including improvements in autonomic regulation and stress adaptation [5]. Mindfulness, defined as the intentional focus of attention on present-moment experiences with a non-judgmental attitude [7], is a meditation-based practice aimed at cultivating awareness of internal experiences and reducing cognitive and emotional reactivity. Its benefits include improvements in psychological well-being, stress reduction, enhanced emotion regulation, and increased health-related quality of life [8,9]. In the context of post-MI recovery, mindfulness-based interventions have shown promise in reducing anxiety and perceived stress, improving blood pressure control, enhancing social support, and supporting overall cardiovascular health [6].

Despite growing interest in yoga- and mindfulness-based interventions within cardiac rehabilitation, several important gaps remain in the literature. Existing studies are often limited by small sample sizes, heterogeneity in intervention protocols, short follow-up periods, and a focus on either physiological or psychological outcomes rather than an integrated assessment. Furthermore, the relative effectiveness of these mind–body approaches compared with standard CR remains unclear, and evidence regarding long-term cardiovascular and psychosocial benefits is still sparse. These limitations hinder the development of clear, evidence-based recommendations for integrating yoga and mindfulness into routine post-MI rehabilitation. Against this background, the present systematic review aims to comprehensively summarize and synthesize the available evidence on yoga- and mindfulness-based interventions in post-MI cardiac rehabilitation. By highlighting both the physiological and psychological effects, and identifying gaps in the current research, this review seeks to inform clinical practice and guide future investigations into integrative approaches for improving recovery and long-term outcomes after myocardial infarction.

## 2. Literature Search and Study Selection

**Figure 1:**
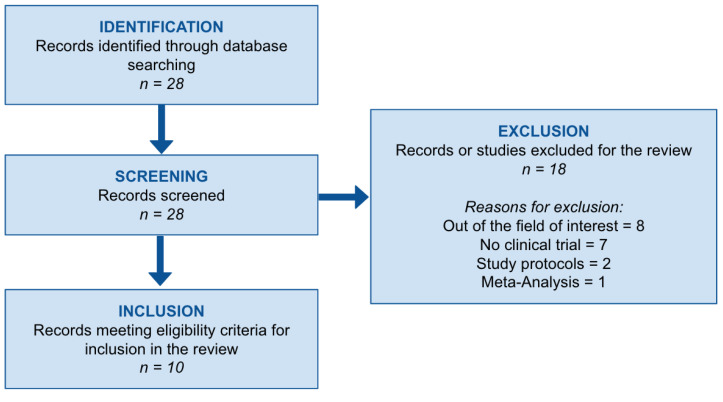
Study flow.

A systematic review was conducted in accordance with the Preferred Reporting Items for Systematic Reviews and Meta-Analyses (PRISMA) guidelines [4] to summarize current evidence on the use of yoga and/or mindfulness-based interventions in rehabilitation after myocardial infarction (MI). A comprehensive literature search was performed across three electronic databases: PubMed, BioMed Central, and the Cochrane Library. The search strategy combined Medical Subject Headings (MeSH) terms and free-text keywords related to myocardial infarction, cardiac rehabilitation, and mind–body interventions. Specifically, the following terms were used: ((“myocardial infarction” [MeSH Terms] OR “myocardial infarction” [Title/Abstract] OR “heart attack” [Title/Abstract] OR “post-MI” [Title/Abstract] OR “post myocardial infarction” [Title/Abstract]) AND (“cardiac rehabilitation” [MeSH Terms] OR “cardiac rehabilitation” [Title/Abstract] OR “rehabilitation” [Title/Abstract] OR “post-MI rehabilitation” [Title/Abstract]) AND ((“yoga” [MeSH Terms] OR “yoga” [Title/Abstract] OR “yogic” [Title/Abstract]) OR (“mindfulness” [MeSH Terms] OR “mindfulness” [Title/Abstract] OR “mind-body” [Title/Abstract] OR “meditation” [Title/Abstract] OR “mindfulness-based stress reduction” [Title/Abstract])). Eligible studies were original research articles published in peer-reviewed journals and written in English, up to October 2025. Manual screening of reference lists of included studies was performed by two independent reviewers to identify any additional relevant articles. Any discrepancies were resolved through discussion and consensus. Both randomized controlled trials and observational studies were considered eligible if they included adult patients with a history of MI undergoing yoga- and/or mindfulness-based rehabilitation interventions. Studies focusing on cardiovascular populations without specific post-MI data, conference abstracts, reviews, editorials, and case reports were excluded. Following the initial database search, 21 records were identified. After title and abstract screening, 11 studies were excluded for predefined reasons, as detailed in the PRISMA flow diagram (Figure 1). The remaining 10 studies met the inclusion criteria and were included in the qualitative synthesis. The methodological quality of the included studies was assessed using the Methodological Index for Non-Randomized Studies (MINORS) criteria through pre-specified electronic evaluation forms [5]. MINORS scores ranged from 10 to 20. No studies were excluded on the basis of quality assessment, given the exploratory and descriptive aim of the present review and the limited number of available studies in this field.

## 3. Effects of Yoga on Cardiovascular Health

**Figure 2:**
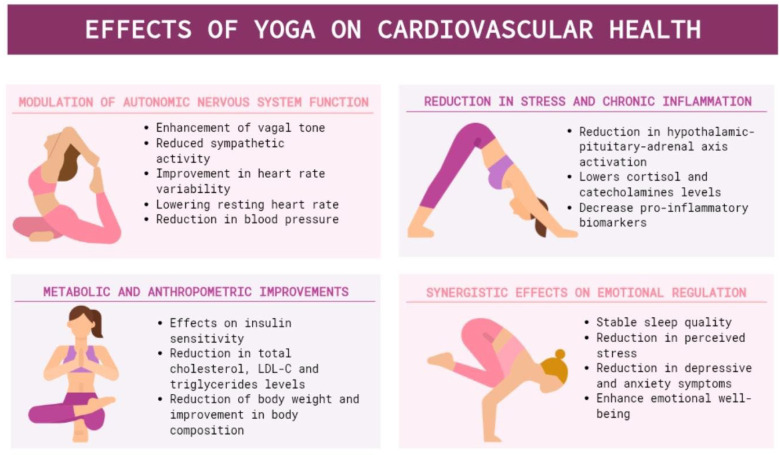
Effects of Yoga on cardiovascular health.

Over the past decades, growing scientific interest has focused on the physiological and clinical mechanisms through which yoga may exert cardioprotective effects (Figure 2). Accumulating evidence suggests that these effects are mediated by the integration of autonomic, neuroendocrine, inflammatory, metabolic, and psychological pathways. One of the most consistently reported mechanisms involves modulation of autonomic nervous system activity. Regular yoga practice appears to shift the sympatho-vagal balance toward enhanced parasympathetic (vagal) tone and reduced sympathetic activation [6]. This autonomic modulation has been associated with improvements in heart rate variability (HRV), lower resting heart rate, and attenuated stress-related cardiovascular responses [7,8]. In parallel, yoga interventions have been linked to modest but clinically meaningful reductions in both systolic and diastolic blood pressure [9,10,11], including in individuals with pre-hypertension [12]. Through sustained stress reduction, yoga may also attenuate systemic inflammation, a key contributor to endothelial dysfunction and atherosclerosis. At the neuroendocrine level, yoga has been shown to reduce activation of the hypothalamic–pituitary–adrenal (HPA) axis, leading to lower circulating levels of cortisol and catecholamines [13]. Consistent with these mechanisms, two systematic reviews have reported significant reductions in pro-inflammatory biomarkers, including C-reactive protein (CRP), interleukin-6 (IL-6), and tumor necrosis factor-α (TNF-α), among individuals practicing yoga [14,15]. Beyond neurohumoral and inflammatory effects, yoga appears to favorably influence several metabolic and anthropometric parameters closely associated with cardiovascular risk. Multiple systematic reviews and meta-analyses have demonstrated modest but consistent reductions in body weight and body mass index (BMI) following structured yoga interventions [10,16]. Improvements in lipid profiles have also been reported, including reductions in total cholesterol, low-density lipoprotein cholesterol (LDL-C), and triglycerides [16,17]. Additionally, a randomized clinical trial observed modest improvements in postprandial glucose levels, suggesting a potential role in glycemic regulation [18]. When integrated within broader lifestyle interventions, these effects may synergistically contribute to improvements in insulin sensitivity, lipid metabolism, and body composition. Yoga has also been associated with improvements in sleep quality, including more restorative sleep patterns and reduced nocturnal awakenings [19,20]. From a psychological perspective, yoga practice has been shown to reduce perceived stress and anxiety, enhance emotional well-being [21], and alleviate symptoms of depressive and anxiety disorders [22,23]. From a cardiological standpoint, evidence in patients with heart failure indicates that yoga may improve exercise capacity—reflected by increases in peak VO_2_ and 6 min walking distance—as well as quality of life, while reducing biomarkers such as N-terminal pro–B-type natriuretic peptide (NT-proBNP) [24]. Long-term clinical outcomes have been less extensively investigated, but notable findings have emerged from a 15-year follow-up study of patients undergoing coronary artery bypass grafting (CABG) who participated in a yoga-based cardiac rehabilitation program [25]. In this cohort, yoga-based rehabilitation was associated with a significant reduction in all-cause mortality compared with standard rehabilitation care (hazard ratio [HR] 0.46, 95% confidence interval [CI] 0.25–0.45, *p* = 0.02), along with a non-statistically significant favorable trend toward reduced major adverse cardiovascular events (MACE) (HR 0.57, 95% CI 0.30–1.04, *p* = 0.065). Among individual MACE components, cardiovascular mortality was significantly lower in the yoga group (HR 0.49, 95% CI 0.24–1.00, *p* = 0.049) [25]. Collectively, these findings support the notion that yoga contributes to cardiovascular health through integrated effects on autonomic regulation, inflammatory burden, metabolic risk factors, and psychological well-being. While most evidence derives from heterogeneous populations and intervention protocols, the consistency of these mechanisms suggests that yoga may serve as a valuable adjunct within the broader spectrum of cardiovascular rehabilitation and prevention, including in patients with established coronary artery disease (CAD).

## 4. Yoga After Myocardial Infarction

Several studies have investigated the role of yoga-based interventions in patients following myocardial infarction (MI), employing heterogeneous rehabilitation protocols (Table 1). Among these, the largest randomized trial evaluated the Yoga-CaRe program, which consisted of 13 supervised, in-hospital yoga sessions delivered over 12 weeks, beginning within two weeks after MI. The first two sessions were conducted individually, followed by group-based sessions, with patients encouraged to continue yoga practice independently at home thereafter. Control participants received enhanced standard care, including educational counseling, alongside guideline-directed medical therapy in both groups [2]. Other smaller trials adopted different intervention formats. In one randomized study, patients assigned to the yoga group participated in supervised yoga sessions three times per week for 12 weeks and were encouraged to practice at home on non-supervised days, in addition to standard pharmacological therapy [26]. Another study compared a 22-day Hatha yoga training program with conventional cardiac rehabilitation [27]. Across all trials, yoga-based rehabilitation demonstrated an excellent safety profile, with no reported adverse events attributable to the intervention. Adherence to yoga practice was generally good in studies reporting attendance, with comparable participation across age groups and sexes [2]. Clinical outcomes varied substantially across studies. Only one trial was powered to explore major adverse cardiovascular events (MACE). In a cohort of 3959 patients with ST-segment elevation myocardial infarction (STEMI), the Yoga-CaRe intervention was associated with a non-statistically significant trend toward reduction in the composite endpoint of all-cause death, non-fatal MI, non-fatal stroke, and emergency cardiovascular hospitalization after a median follow-up of 21.6 months (hazard ratio [HR] 0.90, 95% confidence interval [CI] 0.71–1.15, *p* = 0.41). Importantly, the study was underpowered for the primary outcome due to a lower-than-expected event rate [2]. Effects on cardiac structure and function were inconsistently reported. Sharma et al. found no significant difference in left ventricular ejection fraction (LVEF) following a 12-week yoga-based rehabilitation program compared with standard care [26]. In contrast, Grabara et al. reported greater improvements in LVEF as well as reductions in left ventricular end-diastolic (LVEDD) and end-systolic (LVESD) diameters after 24 days of yoga training, suggesting a potential effect on reverse ventricular remodeling [27]. Psychological and functional outcomes were more consistently favorable across studies. Participation in yoga-based rehabilitation was associated with significant improvements in health-related quality of life and a faster return to pre-infarction daily activities [2]. Reductions in depressive and anxiety symptoms were documented through validated instruments, including the Cardiac Depression Scale (CDS) and the Hamilton Anxiety Rating Scale (HAM-A) [26]. Functional capacity also improved, as reflected by higher Duke Activity Status Index (DASI) scores and derived metabolic equivalents (METs) in patients practicing yoga [26]. Grabara et al. additionally reported significant improvements in peak oxygen consumption (VO_2_), METs, and heart rate at rest and peak exercise following yoga-based rehabilitation [27]. In contrast, effects on traditional cardiovascular risk parameters were less consistent. No significant between-group differences were observed in systolic or diastolic blood pressure across studies [27,28], nor in systolic blood pressure variability or heart rate variability indices in patients undergoing the Yoga-CaRe program [28,29]. A modest reduction in body mass index (BMI) was reported in a European cohort of male patients with STEMI following yoga-based rehabilitation [27]. Improvements in lipid parameters, including reductions in low-density lipoprotein cholesterol (LDL-C), triglycerides, total cholesterol, and the HDL/total cholesterol ratio, along with an increase in high-density lipoprotein cholesterol (HDL-C), were observed in one study, although these changes did not reach statistical significance [26].

## 5. Effects of Mindfulness on Cardiovascular Health

**Figure 3:**
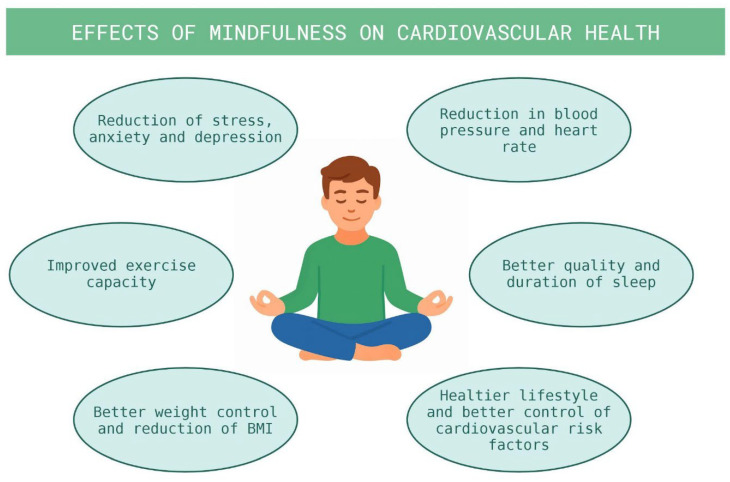
Effects of Mindfulness on cardiovascular health.

Originally rooted in Buddhist contemplative traditions, mindfulness was initially developed to alleviate suffering and cultivate compassion and has progressively gained recognition in the medical field for its application in chronic disease management, particularly in supporting coping with pain and disability [30]. This conceptual framework led to the development of structured mindfulness-based interventions (MBIs), among which Mindfulness-Based Stress Reduction (MBSR), introduced by Jon Kabat-Zinn in the early 1980s, represents the most widely studied model. MBSR is typically delivered as an eight-week program incorporating practices such as body scanning, guided meditation, and gentle yoga. Accumulating evidence has demonstrated its effectiveness in reducing pain intensity, perceived stress, anxiety, and depressive symptoms, while improving psychosocial functioning and health-related quality of life [31,32]. The relevance of mindfulness in cardiovascular medicine is supported by the well-established association between mental health and cardiovascular outcomes. Anxiety and depression are recognized as independent cardiovascular risk factors, linked to increased incidence of major adverse cardiovascular events, higher cardiovascular mortality, and poorer prognosis. Their prevalence among patients with cardiovascular disease is substantial, affecting approximately 18% of individuals with depression and up to 30% with anxiety disorders [33]. These observations provide a strong rationale for interventions targeting psychological distress as part of comprehensive cardiovascular care. The proposed mechanisms through which mindfulness exerts beneficial cardiovascular effects are multifactorial (Figure 3). At a behavioral level, mindfulness may enhance emotional regulation, stress resilience, and self-efficacy, facilitating improved adherence to medical therapy and greater engagement in health-promoting behaviors such as regular physical activity, dietary modification, and smoking cessation [30]. At a biological level, mindfulness practices are thought to modulate central nervous system activity and downstream neuroendocrine and immune responses, primarily through stress reduction. Chronic psychological stress, which is highly prevalent in cardiovascular populations, is known to activate the sympathetic nervous system and stress hormone pathways, promote platelet activation, and impair endothelial function, thereby increasing cardiovascular risk [34,35,36]. Consistent with these mechanisms, recent evidence indicates that mindfulness and meditation practices are associated with modest but clinically relevant improvements in cardiovascular risk factors. These include reductions in systolic and diastolic blood pressure, increased likelihood of smoking cessation, higher levels of physical activity, and improved weight control [37,38]. Meta-analyses and systematic reviews have further reported significant decreases in systolic blood pressure (with an average reduction of approximately 5 mmHg), diastolic blood pressure, heart rate, heart rate variability, and perceived stress levels [39,40,41,42,43,44]. In patients with established cardiovascular disease, mindfulness-based interventions, particularly MBSR, have been associated not only with improvements in psychological outcomes—such as anxiety, depression, sleep quality, and stress—but also with favorable effects on selected hemodynamic and functional parameters. These include reductions in blood pressure and heart rate, improvements in exercise capacity as assessed by the 6 min walking test, and modest reductions in body mass index (BMI) [43,45,46,47,48]. However, the overall quality of evidence remains variable, as many studies are limited by small sample sizes, heterogeneous intervention protocols, and potential sources of bias. Moreover, robust data on hard cardiovascular endpoints are currently lacking. Overall, mindfulness-based interventions can be considered safe, low-cost, and feasible adjuncts to guideline-based cardiovascular prevention strategies. While they should not be viewed as substitutes for established evidence-based therapies, their consistent benefits on psychological well-being, stress reduction, and health-related behaviors support their integration into comprehensive cardiovascular care models.

## 6. Mindfulness After Myocardial Infarction

Psychological distress, including stress, anxiety, and depressive symptoms, is highly prevalent in patients following myocardial infarction (MI) and is independently associated with increased all-cause mortality, recurrent cardiovascular events, and poorer long-term prognosis. In this context, the 2025 European Society of Cardiology (ESC) clinical consensus statement on mental health and cardiovascular disease recommends the use of stress-management strategies, including mindfulness-based interventions, to improve psychological well-being in patients with cardiovascular disease, while acknowledging that their impact on hard cardiovascular endpoints remains uncertain [33]. To date, five studies have specifically evaluated the effects of mindfulness-based interventions in post-MI populations (Table 2) [49,50,51,52,53]. These studies employed heterogeneous intervention protocols, including Mindfulness-Based Stress Reduction (MBSR) [49,50,53], brief Mindfulness-Based Cognitive Therapy (MBCT-brief) [51], and other structured mindfulness-based interventions (MBIs) [52], with program durations ranging from seven days to nine weeks. Interventions were delivered either during the acute post-infarction hospitalization phase or later during recovery. All participants received guideline-directed medical therapy, while control groups were provided with standard lifestyle education in accordance with cardiovascular prevention guidelines. Despite variability in study design and outcome reporting, mindfulness-based interventions consistently resulted in significant improvements in psychological outcomes. Compared with control groups, participants receiving mindfulness interventions experienced reductions in perceived stress, anxiety, and depressive symptoms, along with improvements in sleep quality and duration, health-related quality of life, and illness perception [49,50,51,52,53]. Evidence regarding physiological and clinical outcomes remains limited. In a retrospective study by Gu et al., an eight-week MBSR program was evaluated in 61 MI patients treated with percutaneous coronary intervention and compared with 61 matched controls. The intervention group demonstrated significant reductions in systolic, diastolic, and mean arterial blood pressure, with no significant change in heart rate. Notably, at three-month follow-up, the incidence of major adverse cardiovascular events (MACE) was significantly lower in the MBSR group compared with controls (9.8% vs. 26.2%, *p* = 0.032), although the study was limited by its small sample size and retrospective design [53]. Additional insights derive from a randomized controlled trial conducted by Wu et al., involving 100 MI patients with hemodynamic instability requiring intra-aortic balloon pump (IABP) support. Patients were randomized to receive MBSR in addition to in-hospital cardiac rehabilitation or cardiac rehabilitation alone. The combined MBSR and rehabilitation group exhibited significantly greater improvements in left ventricular ejection fraction (LVEF) and a lower incidence of IABP-related complications compared with the rehabilitation-only group [50]. Taken together, available evidence suggests that mindfulness-based interventions may contribute not only to improved psychological well-being but also to selected aspects of physiological recovery following MI. While the current data are preliminary and heterogeneous, and robust conclusions regarding hard cardiovascular outcomes cannot yet be drawn, the consistency of psycho-cardiological benefits supports further investigation and provides a rationale for the integration of mindfulness into post-infarction care pathways.

## 7. Conclusions

Collectively, the available evidence supports a complementary role for yoga- and mindfulness-based interventions in post–myocardial infarction rehabilitation. Across studies, these approaches have consistently demonstrated benefits on psychological outcomes, including reductions in depressive symptoms, perceived stress, and anxiety, alongside improvements in quality of life and subjective well-being [26]. Given the well-established prognostic relevance of psychosocial factors after MI, these effects are clinically meaningful and address dimensions of recovery that are often insufficiently targeted by conventional cardiac rehabilitation programs. Beyond psychological well-being, selected studies suggest that yoga and mindfulness may contribute to improvements in functional capacity and modifiable cardiovascular risk factors. Reported benefits on exercise tolerance, metabolic efficiency, body weight, and lipid profiles indicate a potential synergy between mind–body interventions and guideline-directed pharmacological therapies in optimizing secondary prevention [45,46,47,48]. Importantly, these interventions were characterized by an excellent safety profile and high patient adherence, supporting their feasibility as adjunctive strategies in routine clinical practice. From a practical standpoint, yoga- and mindfulness-based programs may be reasonably considered in post-MI patients who experience persistent psychological distress, reduced engagement with conventional rehabilitation, or barriers to participation in standard exercise-based programs. Their low cost, minimal infrastructure requirements, and adaptability to both in-hospital and outpatient settings further enhance their applicability, particularly in healthcare systems with limited resources or suboptimal access to comprehensive cardiac rehabilitation. Nevertheless, important limitations remain. The current evidence is largely derived from small, heterogeneous studies that are underpowered to detect effects on hard cardiovascular endpoints. Variability in intervention protocols, duration, and outcome measures limits direct comparability, suggests that these results should be considered preliminary and precludes definitive conclusions regarding optimal implementation strategies. Future research should prioritize well-designed, adequately powered trials with standardized intervention frameworks and longer follow-up, ideally integrating yoga and mindfulness within multimodal rehabilitation models to assess their additive value on clinical outcomes.

In conclusion, while yoga and mindfulness should not be viewed as substitutes for evidence-based cardiac rehabilitation, they represent safe, feasible, and potentially impactful adjuncts that can enrich post-infarction care by addressing psychological, behavioral, and functional dimensions of recovery. Their thoughtful integration into contemporary rehabilitation pathways may help deliver more holistic and patient-centered secondary prevention following myocardial infarction.

## Figures and Tables

**Table 1:** Records included in the review—Yoga approach/intervention.

Selected Records	Patient Number and Characteristics	Intervention Type	Endpoints	Results
Dorairaj Prabhakaran et al. 2020 JACC [2]	3959 patients MI within 2 weeks	Yoga-CaRe ^1^	MACE EQ-5D-5L Return to pre-infarct activities score Tobacco cessation Medication adherence Safety	Significant improvements in self-rated health and pre-infarct activities Positive trend for MACE reduction in underpowered population
K.N. Srihari Sharma et al. 2020 JACM [26]	66 patients MI treated conservatively LVEF 30–50%, NYHA I-II	Yoga every day for 12 weeks at hospital + at home sessions vs control group	LVEF HAM-A, CDS, DASI scores Lipid profile	Significant improvement in stress, anxiety, functional capacity. Positive trend for lipids.
Edmin Christa et al. 2019 IJYT [29]	80 patients post-MI	Yoga-CaRe ^1^	Heart rate variability	N.S.
Edmin Christa et al. 2022 Appl. Psych. Biof. [28]	80 patients post-MI	Yoga-CaRe ^1^	Blood pressure variability	N.S.
Malgorzata Grabara et al. 2020 JCRP [27]	70 male patients MI treated with PCI	Hatha yoga training everyday for 24 days vs standard cardiac rehabilitation	BMI LVEF VO_2_, HR, SBP	Improvements observed in both populations, more remarkable in yoga group. Unbalanced groups.

^1^ Yoga-CaRe protocol: 13 direct gentle yoga practices in 12 weeks, individually and in group, at hospital + at home. HAM-A: Hamilton Anxiety Rating Scale; CDS: Cardiac Depression Scale; DASI: Duke Activity Status Index; HR: heart rate; SBP: systilic blood pressure; LVEF: Left ventricular ejection fraction; N.S.: not significative.

**Table 2:** Records included in the review—Mindfulness approach/intervention.

Selected Records	Patient Number and Characteristics	Intervention Type	Endpoints	Results
Liang et al., 2019, Int J Clin Exp Med [49]	116 patients MI after PCI	MBSR ^1^ 3–5 days after PCI, for 7 consecutive days	Mental states: SAS ^3^, SDS ^4^ Sleep Quality: PSQI ^5^ Satisfaction with Life: SWLS ^6^ Medication Compliance Nursing Satisfaction	Significant reduction in anxiety and depression, significant improvement of sleep quality, patients’ satisfaction with life, medication compliance and nursing satisfaction
Nasiri et al., 2020, JEHP [52]	76 patients post-MI	Mindfulness-based training program 9 weeks	Perceived stress: PSS ^7^ Illness perception: IPQ ^8^	Significant reduction in perceived stress and modify the perception of disease
Gu et al., 2023, BMC cardiovascular disorders [53]	122 patients MI after PCI	MBSR 8 weeks	Hemodynamic parameters, Psychosocial characteristics (PSS, PSSS ^9^, HADS ^10^), quality of life (SAQ-7 ^11^), MACE	No significant HR but significant decreased BP, significant improvement in psychosocial characteristics and quality of life, significant reduction in MACE
Wu et al., 2023, Front. Cardiovasc. Med. [50]	100 patients MI patients assisted with an intra-aortic balloon pump	MBSR, 10–14 session during hospitalization associated with cardiac rehabilitation	Anxiety and depression (SAS, SDS), LVEF, IABP-related complications	Significantly reduced anxiety and depression, improvement of LVEF, less IABP-related complications
Spruill et al., 2025, JACC advances [51]	130 patients post-MI 100% women	MBCT-Brief ^2^	Perceived Stress (PSS), anxiety and depression (HADS, PHQ-9 ^12^), quality of life and disease specific health (SAQ-7, PROMIS-mental ^13^), sleep quality, perceived social support	Improvements in anxiety, quality of life, disease specific health status, sleep duration

^1^ MBSR: Mindfulness-based stress reduction; ^2^ MBCT-Brief: Mindfulness-based Cognitive Therapy brief; ^3^ Self-reporting Anxiety scale, ^4^ Self-reporting Depression Scale, ^5^ Pittsburgh Sleep Quality Index, ^6^ Satisfaction With Life Scale, ^7^ Perceived Stress Scale, ^8^ Illness Perception Questionnaire, ^9^ Perceived Social Support Scale, ^10^ Hospital Anxiety and Depression Scale, ^11^ Seattle Angina Questionnaire 7, ^12^ Patient Health Questionnaire 9, ^13^ PROMIS mental questionnaire.

## Data Availability

No new data were created or analyzed in this study. Data sharing is not applicable to this article.
